# Intermediate to long-term clinical outcomes and survival analysis of the Salto Mobile Bearing total ankle prothesis

**DOI:** 10.1007/s00402-021-03946-5

**Published:** 2021-06-04

**Authors:** C. Stadler, M. Stöbich, B. Ruhs, C. Kaufmann, L. Pisecky, S. Stevoska, T. Gotterbarm, M. C. Klotz

**Affiliations:** 1 Department for Orthopaedics and Traumatology, Med Campus III, Kepler University Hospital GmbH, Johannes Kepler University Linz, Altenberger Strasse 96, 4040 Linz and Krankenhausstraße 9, 4020 Linz, Austria; 2 Orthopaedics and Traumatology, Klinik Diakonissen, Weißenwolffstrasse 13, 4020 Linz, Austria; 3 Orthopaedics, Klinik Diakonissen, Weißenwolffstrasse 13, 4020 Linz, Austria; 4 Ordensklinikum Linz Barmherzige Schwestern, Seilerstätte 4, 4010 Linz, Austria

**Keywords:** Total ankle replacement, Total ankle arthroplasty, Ankle arthritis, Joint replacement, Salto

## Abstract

**Introduction:**

Osteoarthritis of the ankle is a major burden to affected patients. While tibio-talar arthrodesis has been the gold-standard regarding the treatment of osteoarthritis of the ankle joint for many years, at present total ankle arthroplasty (TAA) provides appealing clinical outcomes and is continually gaining popularity.

The aim of this study was to evaluate the intermediate- to long-term clinical outcome including the survival rate of Salto Mobile Bearing TAA (Tonier SA, Saint Ismier, France).

**Material and methods:**

In this retrospective study intermediate- to long-term outcomes measures [Ankle Range of Motion (ROM), American Orthopaedic Foot and Ankle Score (AOFAS score) and survival rate] of 171 consecutive TAA were analysed and compared before and after surgery. Revision was defined as secondary surgery with prothesis component removal, while reoperation was defined as a non-revisional secondary surgery involving the ankle.

**Results:**

At a mean follow-up (FU) period of 7.2 ± 2.7 years (range 2.0 to 14.1 years) there was a significant improvement in ankle ROM (total ROM improved from 25.0° ± 15.0° to 28.7° ± 11.3°, *p* = 0.015; plantarflexion improved from 18.4° ± 11.7° to 20.6° ± 8.2°, *p* = 0.044; dorsiflexion improved from 6.6° ± 5.7° to 8.1° ± 4.9°, *p* = 0.011). AOFAS score increased significantly by 41 ± 15 points after surgery (43.3 ± 11.1 before and 84.3 ± 12.0 after surgery, *p* < 0.001). Overall survival rate within the FU was 81.3% (95% CI 75.3% to 87.3%) with any secondary surgery, 89.9% (95% CI 84.1% to 93.6%) with revision and 93.6% (95% CI 89.8% to 97.3%) with reoperation as endpoint.

**Conclusion:**

This study endorses the previously reported appealing intermediate- to long-term outcomes of the Salto Mobile Bearing TAA. There was a significant increase in ROM and AOFAS score as well as decent implant survival at final FU.

## Introduction

Osteoarthritis (OA) of the ankle is a major burden to many affected patients. It leads to pain and a reduced range of motion (ROM) of the tibio-talar joint. This subsequently impairs mobility and quality of life in general [1, 2]. Patients with end-stage ankle OA show comparable limitations in the quality of life like patients with severe hip OA. In addition to the patients’ physical condition also the psychological condition is affected by OA [3].

In the past, tibio-talar arthrodesis represented the gold-standard for the treatment of end-stage ankle OA [4, 5]. Especially motion-related pain within the tibio-talar joint could be addressed herewith very well [6, 7]. On the other hand, there are side effects like decreased ROM and increased risk of subsequent OA of adjacent joints after fusion of the tibio-talar joint [4, 7–9].

Therefore, many authors promote total-ankle-arthroplasty (TAA) as an attractive alternative to increase function and mobility. While first and second-generation TAA showed poor survival rates and clinical outcomes measures due to a high number of complications [10, 11], current generations of TAA show promising results: several authors reported a reduction in complication rates leading to increased survival as well as improved clinical outcome measures like the American Orthopaedic Foot and Ankle Society (AOFAS) score [12–17]. Hence, TAA gained popularity within the last few years [18–20]. The US-wide share of ankle OA treated with TAA increased from 13% in 2007 to 45% in 2013 [21].

The cementless Salto total ankle prothesis (Tornier SA, Saint Ismier, France) was introduced for clinical routine in 1997. Its anatomically shaped tibial and talar component articulate with a mobile polyethylene insert [22]. Previous studies reported promising results after short-term follow-up (FU). However, there are only a few studies by independent investigators analyzing the long-term results of this type of TAA [14, 22–25]. In addition to that the available numbers of joints investigated in these studies is limited.

Thus, the aim of this study was to evaluate the intermediate- to long-term clinical outcome within a relatively large study population and a long-term follow-up period.

## Materials and methods

### Study population

In this retrospective study, patient records were screened for cementless TAA (Salto Mobile bearing, Tornier SA, Saint Ismier, France), which were implanted consecutively between March 2002 and March 2013 at a University Hospital in Austria. Indications for TAA were primary, posttraumatic or secondary end-stage ankle OA caused by chronic systemic diseases (e.g. rheumatoid arthritis, haemochromatosis), local osteonecrosis or prior infection of the ankle joint. Contraindications for TAA were active ankle infection, insufficient tibial or talar bone stock for example caused by large bone cysts, neurological disorders, poor peripheral circulation, uncontrolled diabetes mellitus and high physical demands in young patients (e.g. physically demanding professions, excessive sports…).

**Fig. 1:**
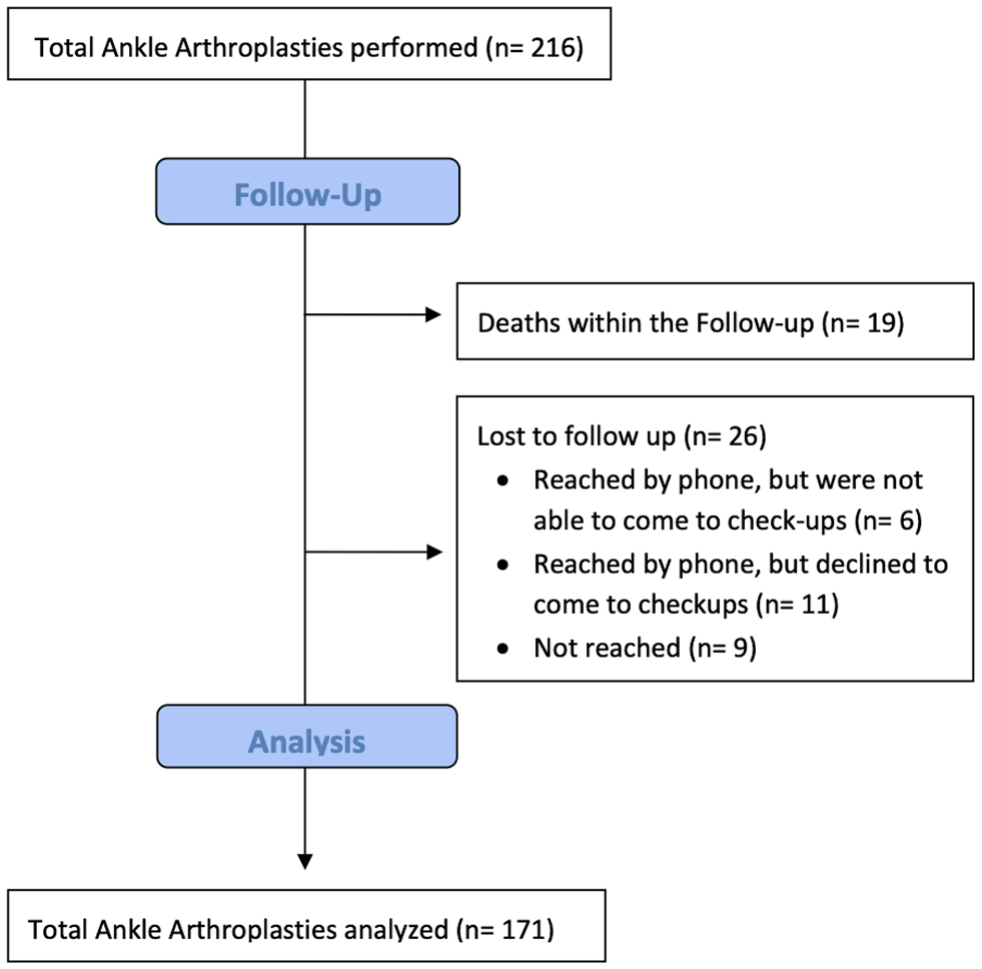
Flowchart regarding the number of deaths within the FU period as well as the number of patients lost to follow up

Within this time period a total of 216 TAA were performed in 212 patients by two senior surgeons each with more than 10 years of experience in foot and ankle surgery. Inclusion criteria were a FU of at least 2 years with at least one postoperative check-up at the outpatient clinic or at least information regarding date and type of revision if any reoperation was performed elsewhere than at the study center. A total of 19 TAA died within the FU period with none of the deaths being related to TAA. In 11 of those cases relatives reported a great satisfaction regarding the prothesis until death while three relatives reported no satisfying results of the TAA with at least one revision surgery of the total ankle prothesis until death and in five cases no relatives of the deceased patient could be reached. 26 TAA were lost to  follow up resulting in an overall study-population of 171 TAA (Fig. 1).

The mean age of the 169 included patients (87 males, 84 females) was 60.5 ± 11.5 years (range: 26.5–87.9 years). The mean FU within our study population was 7.2 ± 2.7 years (range: 2.0–14.1 years).

Indications for TAA were posttraumatic (*n* = 110) or primary OA of the ankle joint (*n* = 36), chronic inflammatory diseases with affection of the ankle joint like rheumatoid arthritis (*n* = 10), prior infection of the ankle joint (*n* = 4), local osteonecrosis (*n* = 3) or chronic systemic diseases with affection of joints (*n* = 2). A combination of posttraumatic and postinfectious OA of the ankle (ankle fracture with subsequent infection) was present in 6 cases. The baseline characteristics of the study population and the indications for  TAA are shown in Table 1 below.

**Table 1:** Baseline demographic characteristics of the study population including the indication for total ankle arthroplasty within the study population

Baseline characteristics of the study population	
Number of patients	169
Number of ankles	171
Mean age (years)	60.5 ± 11.5 (range: 26.5–87.9)
Gender	87 males (50.9%), 84 females (49.1%)
Side	100 right (58.5%), 71 left (41.5%)
Mean body weight (kg)	80.3 ± 14.8 (range: 48–115)
Mean body height (cm)	170.3 ± 8.8 (range: 148–190)
Mean BMI	27.6 ± 4.3 (range: 17.6 – 40.5)
Indications for total ankle arthroplasty	
Posttraumatic osteoarthritis	116 (67.8%)
Primary osteoarthritis	36 (21.1%)
Chronic inflammatory diseases	10 (5.8%)
Prior infection	4 (2.3%)
Aseptic osteonecrosis	3 (1.8%)
Haemochromatosis	2 (1.2%)

### Preoperative examination, operation and postoperative treatment

Preoperatively, a thorough physical examination was performed by a resident or attending from the foot surgery department including the documentation of the AOFAS score and the preoperative active ankle ROM using a goniometer [26]. Informed consent was obtained from all patients. Ethical approval was obtained from the local ethics committee.

Surgery was performed in spinal or general anesthesia according to the recommended standardized technique of the prothesis’ designers using an anterior approach [22, 27]. The calcium hydroxyapatite and titanium coated tibial and talar components were implanted cementless with a mobile ultra-high-molecular-weight-polyethylene inlay providing congruent articulation between those two components. Additional procedures were performed if needed within the primary surgery to address e.g. relevant contractures or bony deficiencies. The most frequent additional procedure performed was Achilles tendon lengthening (*n* = 26), followed by synovectomy (*n* = 18), release of the lateral or medial ligamentous complex (*n* = 13), chiseling of excessive osteophytes (*n* = 8), removement of metal parts from previous surgeries (*n* = 7), talonavicular arthrodesis (*n* = 4), filling of bone cysts (*n* = 3), screw-fixation of intraoperative fractures of the medial malleolus (*n* = 2), talus osteotomy (*n* = 2), osteotomy of the medial malleolus (*n* = 2) and subtalar arthrodesis (*n* = 1).

Postoperatively, a short leg cast was applied to every patient for a total of 6 weeks with no weight-bearing for the first 2 weeks, partial weight bearing for the second 2 weeks and full weight bearing for the third 2 weeks. Venous thromboembolic prophylaxis was provided to all patients throughout that time. After the first 2 weeks, sutures were removed and a new splint was applied. The short leg splint was removed after a total of 6 weeks. Thereafter full-weight bearing was permitted without any limitations, although patients were advised to avoid high-impact activities like long-distance running, mountaineering, playing soccer or tennis.

Physiotherapy was recommended and prescribed to every patient after the splint removal to improve ROM of the ankle joint as well as to regain and improve muscle strength and coordinative skills of the lower extremity.

### Clinical exam and clinical outcome measures

Check-ups at the outpatient clinic took place 2 weeks, 4 weeks, 6 weeks, 3 months and 1 year after surgery. From then on, patients were advised to come to check-ups after every 2 years. This advice was heeded by 56.7% (*n* = 97) of the patients, while 43.3% (*n* = 74) of the patients came to the outpatient clinic for check-ups with more than 2 years between each check-up. The ROM of the ankle was documented within every checkup using a goniometer. The pre- and postoperative AOFAS Score was documented using a printed form.

Data were screened for complications according to the classification introduced by Henricson et al. [28]. Therefore, “Revision” was defined as the removal of one component of the prothesis with exception of an incidental exchange of the polyethylene insert, “Reoperation” was defined as non-revisional secondary surgery involving the ankle and non-revisional secondary surgery not involving the joint was defined as “Additional Procedure”.

### Statistical analysis

SPSS (Version 26.0, IBM) was used for the statistical analysis. Kolmogorov–Smirnov-test was performed to test for normal distribution. As for metric scaled data arithmetic mean value and the standard deviation were calculated and these two parameters were reported as arithmetic mean value ± standard deviation. Wilcoxon Signed-Rank test was used to analyze the significance of the difference between non-normally distributed parameters like the average pre- and postoperative ROM. Kaplan Meier survival analysis was performed to analyze the survival rate of the TAA.

Multiple linear regression analysis was conducted to evaluate the effect of certain patient characteristics such as age, gender, body weight, body height or BMI on the postoperative ROM and the AOFAS score.

Chi-Square test was performed to analyze the significance of the differences regarding the revision rate between certain groups of patients (e.g. posttraumatic OA vs. primary OA). The level of significance was defined at *p* ≤ 0.05.

## Results

### Range of motion

After surgery there was a significant increase in ankle ROM (*p* = 0.015): Mean preoperative ROM was 25.0° ± 15.0° and mean postoperative ROM was 28.7° ± 11.3°. In addition to that mean plantarflexion (18.4° ± 11.7° preoperatively to 20.6° ± 8.2° postoperatively, *p* = 0.044) and mean dorsiflexion (6.6° ± 5.7° preoperatively to 8.1° ± 4.9° postoperatively, *p* = 0.011) showed also a significant increase after surgery. According to the multiple linear regression analysis neither age, gender, body weight, body height nor BMI had a significant effect on the postoperative ROM (Table 2).

**Table 2:** Detailed results of the multiple linear regression analysis regarding the effects of age, female gender, body weight, body height and BMI on the postoperative range of motion of the ankle joint and the postoperative AOFAS score

Variable	Regr.-coefficient	Std.-deviation	*p* - value
Effects on the postoperative range of motion			
Age (years)	0.027	0.096	0.775
Female gender	1.541	2.967	0.604
Body weight (kg)	0.428	1.002	0.670
Body height (cm)	− 0.444	1	0.642
BMI	− 1.165	3	0.686
Effects on the postoperative AOFAS score			
Age (years)	0.133	0.095	0.165
Female gender	0.950	2.845	0.739
Body weight (kg)	− 0.151	0.931	0.871
Body height (cm)	0.153	0.880	0.862
BMI	0.479	2.659	0.857

### AOFAS score

After surgery AOFAS Score increased significantly by 41 ± 15 (43.3 ± 11.1 preoperatively to 84.3 ± 12.0 postoperatively; *p* < 0.001). The detailed evaluation revealed significant improvements in every single sub-section of the AOFAS-Score (Table 3). Again, multiple linear regression analysis showed no influence of age, gender, body weight, body height and BMI on the postop AOFAS Score (Table 2).

**Table 3:** Detailed results of the AOFAS-Score including each subsection of the AOFAS-Score

Outcome measure	Mean preoperative score	Mean postoperative score	*p* - value
Pain	11.9 ± 4.4 (range: 4–30)	33.0 ± 6.7 (range: 10–40)	< 0.001
Activity limitations	3.8 ± 2.1 (range: 0–7)	8.7 ± 1.7 (range: 4–10)	< 0.001
Maximum walking distance	2.1 ± 1.8 (range: 0–5)	4.8 ± 0.7 (range: 2–5)	< 0.001
Walking surfaces	1.3 ± 1.7 (range: 0–5)	3.7 ± 1.4 (range: 0–5)	< 0.001
Gait abnormality	2.9 ± 2.5 (range: 0–8)	7.4 ± 1.6 (range: 0–8)	< 0.001
Sagittal motion	4.6 ± 3.2 (range: 0–8)	5.8 ± 2.6 (range: 0–8)	< 0.001
Hindfoot motion	2.9 ± 2.2 (range: 0–6)	4.0 ± 2.0 (range: 0–6)	< 0.001
Ankle hindfoot stability	7.4 ± 2.2 (range: 0–8)	8.0 ± 0.0 (range: 8–8)	0.001
Alignment	6.6 ± 3.4 (range 0–10)	9.1 ± 2.2 (range: 0–10)	< 0.001
Total AOFAS-Score	43.3 ± 11.1 (range: 10–74)	84.3 ± 12.0 (range: 40–100)	< 0.001

Results are reported in mean score with standard deviation and range in brackets

### Complications and secondary procedures

In a total of 32 cases (18.7%) secondary surgery had to be performed due to complications related to TAA. According to the classification introduced by Henricson et al. [28], 19 of those surgeries (59.4%) were revisions, while 11 of those (34.4%) were reoperations and in 1 of those cases (3.1%) an additional procedure was performed. The indications for secondary surgery are shown in Table 4, while the types of performed procedures are shown in Table 5. In 2 of the cases (6.3%) a revisional surgery was performed in another hospital. For those two cases no detailed information regarding the indication and for one case also no detailed information regarding the type of surgery was available. In 9 cases (5.3%) a combination of two or more complications (e.g. ossification combined with fracture of the inlay) led to revision. As for the revisions with explantation of the prothesis (*n* = 8, 4.7%), a revision TAA was implanted in 3 (1.8%) cases, while arthrodesis was performed in 5 cases (2.9%). Retrograde intramedullary nail arthrodesis was performed in 4 and extramedullary plate arthrodesis in 1 of those cases.

**Table 4:** Detailed numbers regarding the complications that led to secondary surgery within the study population

	Overall (*n* = 32)	Revision (*n* = 19)	Reoperation (*n* = 11)	Add. procedure (*n* = 1)
Soft tissue impingement	9	5	4	
Periarticular ossification	8	4	4	
Inlay fracture	7	7		
Osteolytic cysts	5	3	2	
Wear	4	4		
Acute infection	2	2		
Aseptic necrosis	2	2		
Instability	1	1		
Achilles’ tendon rupture	1			1
Talonavicular OA	1		1	
No information available	2	1

**Table 5:** Detailed numbers regarding the types of surgeries that were performed secondary to TAA due to complications within the study population

	Overall (*n* = 32)	Revision (*n* = 19)	Reoperation (*n* = 11)	Add. procedure (*n* = 1)
Synovectomy	17	12	5	
Achilles’ tendon lengthening	2		2	
Achilles’ tendon repair	1			1
Lateral ligament repair	1	1		
Removal of ossifications	9	5	4	
Filling osteolytic cysts	5	3	2	
Talonavicular arthrodesis	1		1	
Inlay replacement	22	11	11	
Explantation	8	8		
Arthrodesis	5	5		
Revision prothesis	3	3		
No information available	1			

The mean time from index surgery to revision was 5.8 ± 3.3 years, while the mean time to reoperation was 4.0 ± 2.1 and the additional procedure was performed 2 months after index surgery.

There were no significant differences regarding the rate of secondary surgeries (including all revisions, reoperations and additional procedures) when comparing patients with posttraumatic arthritis (22 secondary surgeries out of 116 patients; 18.9%) to patients with primary arthritis, chronic inflammatory diseases or other indications for total ankle arthroplasty (10 secondary surgeries out of 55 patients; 18.2%) within our study population (*p* = 0.945). Also, there was no significant difference regarding the mean time from index surgery to secondary surgery (posttraumatic OA: 5.4 ± 2.9 years, other indications: 4.25 ± 3.4 years; *p* = 0.341).

### Prothesis survival analysis

**Fig. 2:**
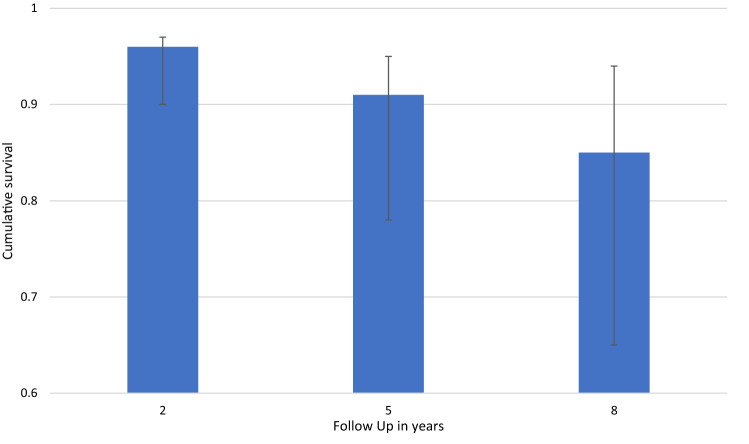
Survival rates within the follow up after 2, 5 and 8 years with any secondary procedure as endpoint. The error bars show the 95% KI

The overall survival rate of the prothesis within the FU period (Mean: 7.2 years; range: 2.0 to 14.1 years) with any secondary surgery as endpoint (including all revisions, reoperations and additional procedures) was 81.3% (95% CI 75.4% to 87.2%). Figure 2 shows the according survival rates after 2, 5 and 8 years. The survival rate with revision as endpoint was 89.9% (95% CI 84.1% to 93.6%) while the survival rate with reoperation as endpoint was 93.6% (95% CI 89.8% to 97.3%) within the FU period of our study population.

## Discussion

The results of this study show a slight but statistically significant improvement of 3.7° in ankle total ROM (*p* = 0.015) after TAA. This matches the findings of other authors who reported an increase in ROM after implantation of the Salto total ankle prosthesis. Nevertheless, in most of their studies a higher increase in ROM after TAA was reported [22, 24, 25]. In this study, plantarflexion increased from an average of 18.4° preoperatively to 20.6° postoperatively (*p* = 0.044) and dorsiflexion increased from an average of 6.6° preoperatively to 8.1° postoperatively (*p* = 0.011). These findings are in contrast to previous studies which reported just a significant improvement in dorsiflexion but not in plantarflexion after implantation of the Salto Mobile Bearing TAA [22, 25]. In this study, patients were permitted no weight bearing for 2 weeks after surgery. Afterwards partial weight bearing was allowed from week 2 to 4 and full weight bearing from week 4 to 6 after surgery. During that time the ankle was immobilized in a cast. The duration of immobilization and the amount of weight bearing might influence postoperative ROM and differs significantly in the literature. For example Bonin et el. permit full weight bearing immediately after surgery [29] while Schenk et al. permit progressive weight bearing 8 days after surgery and full weight bearing 10 days postoperatively [24] and Koo et al. permit no weight bearing for the first 2 weeks after surgery [23]. Additionally, both pre- and postoperative ROM within this study were measured using a simple goniometer at the outpatient clinic. Therefore, there might be a measurement bias, which limits the validity of our findings. Dekker et al. reported, that clinically measured ROM of TAA tends to be overrated by up to 12° caused by increased subtalar and midfoot ROM after TAA [30]. For future investigations the accuracy of the measurement of the ROM could be improved for example by using a special radiographic technique [31]. Eventually, despite its statistical significance, the clinical relevance of the average ROM-improvement after TAA found in this study remains questionable.

In this study, the AOFAS-Score increased significantly by 41 ± 15 points after surgery to an average of 84.3 ± 12.0 points at final follow-up. This matches the findings of many other studies regarding the Salto Mobile Bearing TAA as well as other models of TAA and underlines the high patient satisfaction after TAA [22, 23, 32, 33, 34]. For instance regarding Salto Mobile Bearing TAA Schenk et al. reported an average AOFAS-Score of 82.2 ± 14.0 points at final follow-up [24], while Wan et al. reported an average AOFAS-Score of 80.2 ± 15.3 points at final follow-up [25] and Faber et al. reported an average AOFAS-Score of 85 ± 5 points at final follow-up [14]. Including other models of TAA as well, a meta-analysis conducted by Onggo et al. reported an average improvement of 43.60 points (95% CI 37.51 points to 49.69 points) after TAA [15].

The most frequent complications within this study were soft tissue impingement followed by periarticular ossifications, fracture of the polyethylene inlay and osteolytic cysts. All of those complications have been reported in previous studies regarding TAA mostly in a comparable incidence [29, 34–38]. In our study population, pain or limited mobility caused by soft tissue impingement led to secondary surgery in 5.3% (*n* = 9) of all cases. This matches the findings of Kim et al. who reported soft tissue impingement that led to secondary surgery in 5.8% of their cases [37]. Symptomatic periarticular ossifications after TAA led to secondary surgery in 4.7% (*n* = 8) of all cases, which is also mostly in line with available publications addressing this complication. For example, a systematic review conducted by Bemenderfer et al. reported an average rate of secondary surgeries caused by periarticular ossifications of 7.2% with rates ranging from 0 to 34.2% in available literature [39]. Inlay fracture led to revision in 4.1% (*n* = 7) of the cases within the follow-up period. Although inlay fracture was one of the top three indications for secondary surgery in general and the top reason for revision of the prothesis itself, the rate of inlay fracture found in this study is slightly lower when comparing it to a publication of Labek et al., who analyzed worldwide TAA registers and found rates of inlay fractures ranging from 5 to 10% [40].

The overall survival-rate with any secondary procedure as endpoint (including revisions, reoperations and additional procedures) was 81.3% within the FU (Mean FU: 7.2 years; range: 2.0 to 14.1 years). The prothesis-survival-rate with revisions of the prothesis as endpoint was 89.9% respectively 93.6% with reoperation of the ankle as an endpoint within the same period. In comparison to available data regarding the Salto Mobile bearing TAA, our findings showed a lower survival rate with any secondary procedure as endpoint (81.3%) compared to other previous studies (e.g. Koo et al.: 90.2%, Wan et al.: 88.1%) [23, 25, 41]. Bonin et al. reported a survival rate of 91.8% with revision or radiographic loosening as endpoint after a mean FU of 2.9 years. At a mean FU of 8.9 years their survival rate decreased to 85% with fusion or revision of at least one component of the prothesis as endpoint, respectively, 65% with any reoperation as endpoint. [22, 29]. In addition, Koo et al. reported a drop of the survival rate from 90.2% with revision or reoperation as endpoint at five years to 86.2% from 6 to 10 years [23]. Therefore, the endpoint of final FU and evaluation of long-term survival is important, as the data mentioned above suggests a significant increase in revision rates over time. In comparison to many other studies, our study provides a relatively long average FU period in regard to a comparatively large study population.

In this study population, there was no difference regarding the incidence of revisions when comparing patients with posttraumatic OA to patients with other indications for TAA. This does not agree with other reports, which showed higher revision rates in patients with posttraumatic osteoarthritis of the ankle [24, 36, 42]. Although Schenk et al. [24] as well as Gramlich et al. [36] interestingly investigated mostly the same type of TAA.

There are several limitations of this study that have to be mentioned: First, this is a retrospective study. This limits the level of evidence as a prospective study would be a more appropriate design to investigate the outcomes analyzed in this study. Second, the ROM reported within this study was documented using a simple goniometer and therefore might not be as accurate as ROM outcomes measured with radiological techniques [30]. Third, only a single clinical outcome measure (AOFAS-Score) was evaluated within this study. Additionally, the AOFAS-Score is no validated score and is not recommended for clinical usage anymore [43, 44]. Regarding the statistical analysis, the study population is relatively small for some of the performed tests (e.g. multiple linear regression analysis). Therefore, the results of this study have to be interpreted with some caution. This represents another limitation of this study.

Nevertheless, the study presents intermediate- to long-term results (mean FU > 7 years) of a large study population (169 patients/171 TAA) compared with available publications [14, 22–25, 29, 41]. The study was conducted by independent investigators at a university hospital and received no external funding. Hence, this study adds further relevant information regarding mobile-bearing TAA to the literature available to date and—keeping the limitations mentioned above in mind—suggests that Salto Mobile Bearing TAA is a reliable treatment option for end-stage ankle OA with decent intermediate- to long-term clinical outcomes and implant survival.
